# Perceptions, emotional reactions and needs of adolescent psychiatric inpatients during the COVID-19 pandemic: a qualitative analysis of in-depth interviews

**DOI:** 10.1186/s12888-021-03378-w

**Published:** 2021-07-28

**Authors:** George Giannakopoulos, Savvina Mylona, Anastasia Zisimopoulou, Maria Belivanaki, Stella Charitaki, Gerasimos Kolaitis

**Affiliations:** Department of Child Psychiatry, School of Medicine, National and Kapodistrian University of Athens, “Aghia Sophia” Children’s Hospital, Thivon and M. Assias, 115 27 Athens, Greece

**Keywords:** Adolescents, COVID-19, Mental health, Pandemic, Psychiatric inpatient unit

## Abstract

**Background:**

The new coronavirus pandemic (COVID-19) has been accompanied by severe psychological pressure on the entire population. However, little is known about how this pandemic could affect the more vulnerable population with severe mental illness.

**Aims:**

To explore adolescent psychiatric inpatients’ perceptions, emotional reactions and needs during the first wave of the COVID-19 pandemic.

**Methods:**

Individual in-depth interviews were conducted with nine psychiatric inpatients aged 12–17 years. Through open-ended questions, interviewers initiated five themes: (a) knowledge about coronavirus pandemic, (b) changes in everyday routine due to the pandemic, (c) adolescents’ feelings about the pandemic, (d) adolescents’ positive thoughts and behaviors, and (e) how the social environment can help adolescents deal with the pandemic-related situation. A thematic analysis was conducted using line-by-line open coding.

**Results:**

Regarding their knowledge of the impact of the current pandemic, almost all adolescents focused on information about the nature of coronavirus and on existing crisis management practices. Almost all patients identified predominantly negative changes due to the quarantine state, including restrictions on both social life and personal freedom as well as excessive contact with family members during home isolation. As far as their emotions were concerned, adolescents did acknowledge anxiety about self-harm and harming their loved ones as well as mood swings within the family nucleus; anxiety was also manifested about the unknown and the management of the pandemic in other countries. Avoidance of thought rumination about the coronavirus and its consequences, positive thinking and looking towards the future were reported as constructive strategies for coping with challenging emotions. Additionally, a sense of belongingness seems to have been playing a pivotal role in the adolescents coping strategies. Trust in the authorities and the community was another quite noteworthy point that emerged during the interviews. Lastly, our findings indicated adolescents’ benefit from receiving balanced health messaging coupled with balanced thinking within their social and family environment.

**Conclusions:**

Enhanced comprehension of possible mediating psychological pathways is needed to help clinicians, researchers, and decision-makers to avert the deterioration of mental disorders and overall functioning, as well as additional stress-related disorders.

**Supplementary Information:**

The online version contains supplementary material available at 10.1186/s12888-021-03378-w.

## Background

The first wave of the new coronavirus pandemic (COVID-19) struck the entire world in the first quarter of 2020 with many countries still struggling to mitigate its magnitude on public health through strict quarantine measures, social distancing and health education strategies [1]. This unprecedented phenomenon is equivalent to a mass disaster. Mass disasters affect individuals, families and entire societies and typically result in major physical, psychological, and economic consequences. The overall and enduring impact of a mass disaster may disrupt societal networks of children and their families alike, daily life routines and also question all sense of security and cohesion [2]. Under the pandemic threat, society’s ability to facilitate the recovery of its members is being dramatically reduced. The situation is aggravated as resources run out and health, education and social welfare facilities are being compromised.

During the ongoing pandemic, morbidity, and mortality aside, psychological pressure on the entire population is particularly high [3]. Fear of infection and its outcomes, loneliness, denial as well as anxiety and obsessive-compulsive disorder symptoms, ennui, frustration, irritability, depression, and despair may be experienced by a significant part of the population [4–8]. In addition, the ensuing economic crisis and job loss coupled with social rejection, discrimination, and stigma, further jeopardize mental health [9].

There are numerous mental health risks for children and adolescents which are pandemic-related [10–14]. During pandemic’s acute phase of the pandemic, social distancing, increased pressure on families and minimized access to support services have been observed. Following the pandemic, mental health services have been asked to tackle issues such as economic recession and anxiety, stress, and violence exposure [15, 16].

Patients with pre-existing severe mental illness are inevitably affected by the aforementioned circumstances, thus experiencing more intense discomfort [17, 18]. By way of illustration, patients in psychiatric intensive care units may experience the increased threat of a group infection [5], while psychosocial impact on children and adolescents in inpatient psychiatric units is likely to be much more critical [19].

Youths’ experiences during a disaster like the current pandemic may usually be classified under three inter-related patterns: (a) disruption of normality, (b) fear due to an either objective or subjective threat to life as well as due to the exposure to fear-provoking information, and (c) mourning following a loss (be it the loss of human life or a sense of meaning as well as of basic trust and/or self-esteem) [2]. However, the inner experience of children and adolescents may be overlooked even by the most sensitive observers. Therefore, clinicians and researchers should take into consideration and evaluate the type, extent, and consequences of children’s and adolescents’ emotional and behavioral responses. Moreover, children and adolescents with pre-existing severe mental health problems belong to a high-risk group for worse outcomes not least because of disrupted access to mental health services and impairment of the ability for adaptive coping and emotion regulation [15, 20–22].

The aim of our research was to qualitatively investigate, through in-depth interviews, the perceptions, emotional reactions and needs of adolescent psychiatric inpatients amidst COVID-19 during the second week of April 2020. The first confirmed COVID-19 case in Greece was reported on February 26, 2020 and by March 23 strict containment measures had been adopted by the government to manage the initial wave of the pandemic, including: (i) a national lockdown that restricted all but essential movement and economic activity, (ii) school closures, (iii) domestic travel restrictions, (iv) travel bans on visitors from high-risk countries; and (v) quarantines for international visitors and Greek nationals returning from abroad. At the time of the data collection approximately 100 total coronavirus deaths and 2200 active cases had already been recorded [23].

The method of in-depth interviews offers the opportunity to collect detailed information regarding the comprehension, thoughts and feelings of hospitalized children and adolescents with severe psychiatric disorders. It is hoped that the analysis in question will provide preliminary insight regarding this vulnerable population in the face of an unprecedented disaster. The findings of the present research could enable clinicians, researchers and decision-makers develop psychosocial support interventions as well as prevent not merely the deterioration of mental disorders and overall functioning but also additional stress-related disorders.

## Methods

### Participants and procedure

The study included all nine inpatients aged 12–17 years that were treated during the second week of April 2020 in the psychiatric intensive care unit of the Department of Child Psychiatry, School of Medicine, National and Kapodistrian University of Athens, “Aghia Sophia” Children’s Hospital. Hence, the study employed a purposive (total population) sampling no saturation point was used to determine the sample size and data collection. Participants 1, 2, 4, 5, and 9 were discharged from hospital to home sheltering and continued their treatment program via Skype (individual supportive psychotherapy, parent counselling and family sessions), while the remaining adolescents (Participants 3, 6, 7, 8) continued in-patient treatment. In this setting’s modification, the first group of adolescents was interviewed via Skype, while the second group in vivo, in the psychiatric intensive care unit premises. Participants were treated for a range of disorders, such as anorexia nervosa, obsessive-compulsive disorder, major depressive disorder, autism spectrum disorder, intentional self-harm, and generalized anxiety disorder.

Individual in-depth interviews were conducted with every patient by one female interviewer with appropriate 5-h training in the interview guide. The second and the third author, who were working as child and adolescent psychiatry trainees in the psychiatric intensive care unit at the time of the study, conducted the interviews. Prior to the final administration, an in-depth interview guide was developed, pilot tested and reviewed. The average duration of each interview was approximately 20 min. No one else was present besides the participants and researchers during the interviews. The participants were given a brief description of the current situation pertaining to the COVID-19 pandemic and were subsequently asked to participate in the discussion. They were then asked an open set of questions related to the aims of the present research: *(a) What do you know about the new coronavirus? (b) How has your daily life changed due to the coronavirus? (c) How do you feel about what have been happening? (d) Is there anything you can think or do to feel better? (e) Is there anything other individuals can do to help you feel better?* The questions were developed for this study and have not previously been published elsewhere.

All the in-depth interviews were conducted, audio-recorded and verbatim transcribed in Greek. The transcripts were not returned to participants for comment and/or correction. No field notes were made during the interviews and no repeat interviews were carried out.

Participants were informed of the study aims and objectives. In addition, it was highlighted that participation in the study was voluntary and all information would be treated confidentially.

### Data analysis

A thematic analysis using line-by-line open coding was conducted to explore the understanding, thoughts and emotions of children and adolescents treated in child and adolescent psychiatric inpatient units [24, 25]. The concepts generated by the raw data were then grouped into conceptual categories. Constructed codes were created from in vivo codes (the exact wording used by participants in the interviews). Constant comparisons for developing themes and seeking data not conforming to each theme were carried out by two researchers independently. At the end of the process, no new information was extracted and repetition of information in each of the categories was taken into account. The analysis was both inductive and deductive. More specifically, while responses were open-coded and the analysis was driven by data, they were ultimately grouped into wider categories based to some extent on the interview questions. Participants did not provide feedback on the findings.

## Results

The analysis included the themes that were initiated by interviewers through the five open-ended questions: (a) knowledge about coronavirus pandemic, (b) changes in everyday routine due to the pandemic, (c) adolescents’ feelings about the pandemic, (d) adolescents’ positive thoughts and behaviors, and (e) how the social environment can help adolescents deal with the pandemic-related situation. Each theme is reported below, **and all conceptual categories are highlighted in bold**, while examples of their contents are illustrated with participants’ quotes that were translated from Greek after the completion of the thematic analysis.

### Knowledge about the coronavirus pandemic

**Information about the nature of the coronavirus** was the most common answer between the participants concerning the coronavirus pandemic, including the definition of the coronavirus, its worldwide spread rate, the morbidity, the transmission risk/rate as well as the mortality, outcomes and past knowledge about the pandemic and the origins of the virus.

*many people have died from all this … the older ones can easily die…* (Participant 1)

*others go through it a little heavily, others more lightly … I know that mostly older people get sick … children fortunately go through it very lightly … because, unfortunately, it is very easily transmitted by children to adults mainly* (Participant 8)

A group of participants commented on the efforts of **combating coronavirus pandemic** (i.e., existing crisis management practices in Greece and on the existing knowledge about treatment and disease prevention):

*In Greece we have handled it quite well, so things are better than in other countries that have been hit harder than us* (Participant 1)

*… unfortunately, it has not yet been found, at least to the best of my knowledge, either a drug or a vaccine or anything else … we hope to find a vaccine* (Participant 2)

Additionally, one participant referred to the **psychosocial aftermath** of the pandemic, reflecting on the consequences for infected people and their social environment:

*The (infected) are afraid of the pandemic and feel anxiety, isolation ... they can't see their loved ones ... even their friends, their relatives... Very sad indeed* (Participant 9)

### Changes in everyday life

Initially, three main conceptual categories emerged from the participants’ interviews referring to changes in everyday life: **positive changes**, **negative changes,** and **non-important changes.**

Regarding the **positive changes**, only two participants acknowledged certain positive aspects of the pandemic’s impact on daily life. Specifically, these aspects comprised of the precautionary measures, the strict measures as well as their necessity and the observed compliance of the population to them. What is more, a sense of transience was recorded. Having said that, all the above is to be considered more as helpful attitudes and beliefs that enable the participants cope with his pandemic-induced stress, rather than through substantially positive aspects of the pandemic.

*... unless we consider the general condition worldwide, the strict measures that each country has taken... I understand these measures that have been taken and I believe all this is happening for our own good, so I comply with them like any other citizen … and I believe that all this will pass, and we will continue our daily routine as before...* (Participant 2)

A second non-hospitalized participant viewed the increased contact with loved ones during home restriction as a positive aspect of the pandemic:

*... so, we are all together again... it’s been a long time since we’d all been together like that... it was an opportunity to spend some time together...* (Participant 1)

Almost all participants mentioned **negative changes** in everyday life. Limitations on social life were the most referred to as a negative change. More specifically, nearly all participants referred to the decreased contact with loved ones, the restrictions on socializing opportunities as well as the indoors confinement and schools’ closure.

... I cannot meet my loved ones in person... (Participant 1)

On the contrary, one non-hospitalized participant recognized a negative aspect related to the increased contact with loved ones.

*... there can sometimes be disagreements or fights about many issues...* (Participant 1)

Another particularly interesting concept that emerged from quite a few non-hospitalized participants' responses was that of restriction of personal freedom and prohibitions.

*... obviously I can't go out whenever I want to* (Participant 1)

*… we can only go out when... for some important reason... and still one person at a time...* (Participant 2)

*... we must send a text message if we need to go out...* (Participant 6)

Regarding the adaptation of new obligatory protective behaviors, some participants referred to new hygiene rules that must be followed and to a necessary modification of social encountering.

*We must be within distance from each other, we don't hug each other too much …* (Participant 3)

Finally, some hospitalized participants did not acknowledge any significant changes in everyday life, in view of their pre-existing confinement in the inpatient unit before the onset of the pandemic.

*Ehm... of course in here, ok, things have changed very little ...* (Participant 6)

### Feelings about the pandemic

Regarding participants’ feelings about the pandemic, four main conceptual categories emerged from their answers: **negative feelings**, **positive feelings**, **ambivalence**, and **non-important changes in feelings**.

Nearly all participants expressed negative feelings about the pandemic. Half of the participants expressed worries about possible harm to their loved ones, without overlooking some mood changes within the family nucleus, while possible harm to self.

was referred to a much lesser extent by participants.

*I feel a bit anxious because my mum unfortunately belongs to a vulnerable group ...and I want to know that she is well, all the time... I am very concerned about my grandparents who are old... about the people whom I love, and I care about* (Participant 8)

*I see my brother, who is a student and obviously used to go out very often, being more irritable and getting mad more easily...* (Participant 1)

*... but ok, I can’t say I am really worried about contracting it myself...* (Participant 1)

Some participants expressed concern about the future and the unknown:

*I’m worried about how things will evolve – I mean, how am I to go to school next year ... what will happen with the exams ... all this stresses me out ... I like to know my schedule and not be, like, in a "wait- and-see" situation...* (Participant 1)

*I feel anxiety about when this thing will come to an end …* (Participant 6)

Notably enough, two participants raised concern about the management of the pandemic from a global perspective:

*In other countries, cases are either increased daily or are way too many...* (Participant 2)

Few participants expressed sadness about the deceased or affected people in general. However, more expressed their sorrow regarding the lack of contact with their loved ones, while sadness was also associated with anger and ennui:

*I’m sad about all these people who have died, or those who are ill, and we still don’t know their outcome …* (Participant 2)

*I also feel very bad about the other people who used to be free before but now are all homebound...* (Participant 8)

*I feel a bit sad for not being able to see my dad, because - as a high-risk individual - he is not allowed to get around...* (Participant 3)

*I also feel sorrow that I do not see my family...* (Participant 9)

*I feel sadder because I’m mainly bored...* (Participant 6)

*... I simultaneously feel anger... and maybe sadness...* (Participant 9)

Four of the participants emphasised on the feeling of being confined (‘trapped’, even) and of having been deprived of their freedom. Some of them also said that hospitalisation has been keeping their routine unchanged, whilst others as an aggravating factor of their feelings of confinement and loneliness.

*I don’t feel very well ... but I manage. Everybody must be feeling like that, like they are ‘boxed’ in a house* (Participant 5)

*... It would help me to be out, not in here... to be with my brothers, my family, with the ones I love. ... I cannot do the things I want when I’m shut in here, and I feel like being on my own.* (Participant 7)

Notably, positive feelings about the pandemic were also recorded between the participants. Several participants expressed optimism, while participants who were subjected to home isolation did acknowledge some positive feelings about the lockdown due to the increased amount of time spent with their loved ones.

*This will not be for too long, now that the temperature is rising and it’ll be warm, it will go* away (Participant 3)

*...on the other hand, I’m happy to hear on the news that cases are decreasing daily, at least in Greece... I believe everything will be fine...* (Participant 2)

*I enjoy the days spent with my parents, because I know that when we go back to normality my mum will be working work until late, my dad will return to his shifts and all that... so it was a chance for all of us to be together* (Participant 1)

Finally, when asked about their feelings about the current situation, some inpatients were in two minds. Mixed feelings aside, two participants expressed non-significant changes in their emotions.

*I feel like the other times... ok. I can play with my sisters and watch TV* (Participant 4)

### Helpful thoughts and behaviors

Initially, from the participants’ interviews about what they could do to feel better two main conceptual categories emerged, namely **helpful thoughts** and **helpful behaviors**.

Some participants referred to the avoidance of ruminating about the pandemic as a helpful mindset during the pandemic. In addition, it appears that focusing on the positive aspects of the current situation, such as the low morbidity- mortality rates in Greece and knowing that their loved ones are healthy, is a helpful coping mechanism, Another particularly interesting finding was that many participants were helped by focusing on what the future brings - by maintaining, in other words, that this is merely a transient situation; looking forward to resuming their daily routine and seeing the end of restrictions whilst making plans for the future, also seem to be helpful. Moreover, two participants acknowledged the feeling that they are not alone, in the sense that this is a commonly shared experience. This later mode of thinking also seems to be a comforting one that helps them cope with their worries.

*… and generally, to not feel as if I'm alone in all this …* (Participant 1)

Finally, regarding **helpful thoughts**, a crucial factor is that adolescents put trust in both the authorities and in the community. Indeed, many participants positively commented on the solid operation of Greek state’s institutions and on the citizens’ compliance with the measures. Trust in the scientific community was also reported.

*… and I think that Greece has taken preventive measures much earlier, compared to other countries, and [I think] that, at present, we are one of the safest countries* (Participant 6)

… *there haven’t been many violations* (Participant 1)

*... we have taken precautions ... and that reassures me*... (Participant 6)

*... both doctors and experts do the best they can to help people feel safe...* (Participant 2)

On the issue of **helpful behaviors**, few participants admitted that regulating the amount of information received is of paramount importance. More specifically, two participants voice their need to hear positive news about the pandemic so that the former may feel better. One non-hospitalized participant focused on his need for minimal media information whilst emphasizing their constant need to be kept informed about what has been going on.

*I generally don't sit and watch the news all the time … this doesn’t help me obviously … I watch very little, just enough so as not to live in a bubble* (Participant 1)

Additionally, almost all participants mentioned a need for imaginative leisure activities alongside the need to engage with others through contact with loved ones as well as by resorting to them for comfort when necessary:

*Obviously, talking to my friends and not isolating myself helps; the same goes for talking with my grandparents via Skype as much as I can .... and, when I'm feeling anxious, I visit my parents for support ... I believe in this...* (Participant 1)

### How the social environment can help adolescents deal with the pandemic-related situation

Some participants found information overload to be unhelpful. Instead, adolescents pointed out that it is necessary to calibrate the flow of information received. Nevertheless, they did acknowledge that they need to be kept in the know about the latest developments regarding the pandemic **(balanced health messaging)**. Additionally, some participants thought that adults’ willingness to answer their queries and provide positive updates was beneficial to them.

*Obviously [I want the family] to not hide things from me, to not tell me ‘everything is fine’, because, ok, I wouldn’t like to not know what is going on... but I wouldn’t like to hear them overanalyzing all this with aimless discussions either* (Participant 1)

**A balanced approach and mindset in the environment** were identified as a helpful strategy by several adolescents, especially by those who focused on their family’s positive attitude and avoidance of excessive panic:

*… if a family’s or the relative’s, or the wider environment’s general perception is positive and correct, then the child does not worry that much and feels relief* instead (Participant 2)

*Just others to not feel sad and not give up* (Participant 5)

*To not exaggerate... to not act as if this is the end of the world …* (Participant 1)

What is more, half of the participants considered **emotion regulation within the environment** to be helpful. An emotionally serene atmosphere wherein all family members can manage stress and express painful feelings with a view to receive reassurance through fruitful discussion was also a desideratum.

*... that tensions will be not... and others will not be stressed, because stress is transmittable, so when my parents feel anxious, I can sense it too* (Participant 1)

*... that I could talk to my parents or my sister about the problems that worry me* (Participant 9)

*... and they would tell me things like “this will go away soon”, “it will not last too long, and then things will be like they used to be’* (Participant 3)

A **family’s positive emotional climate** proved to be the most reported helpful aspect for most of the adolescents. This in turn suggests that family would offer ample room for discussion as well as display empathy so that the children can feel a closeness with others and that they can be given support, escaping thus loneliness. In a similar vein, some participants reported the physical proximity to their loved ones as quite a helpful parameter.

*When, for example, my relatives discuss with me, this helps me a lot (Participant 6)*

*... and when, to a great extent, they understand how we feel* (Participant 2)

*What I need now, I believe, is for my family to stand by me... because I don’t feel alone in that way* (Participant 1)

*It would help me very much, if the hospital staff could pressurise the institution staff to come and get me... as soon as possible... and then, if they could bring my brothers here to see them for a bit, but they can’t actually consider where they live...* (Participant 7)

Another helpful way of dealing with the coronavirus pandemic, as mentioned by two non-hospitalized participants, was the stimulus for **shared leisure time and distractions** from others.

*... to spend our time creatively, to not have many moments that I would sit and think on my own about what will happen... so, when others are by me and draw my attention away from all this coronavirus issue, I believe this helps me a lot* (Participant 1)

*... when I discuss together with the people with whom I share the same space, this helps take our minds off all this* (Participant 6)

One participant talked about **looking into the future** (i.e., plans for the future and the removal of restrictions) as being a comforting expectation.

*... it helps for others to tell me several things we can do and arrange when the pandemic ends, for example, discussing where we can go out, or where to spend the summer holidays* (Participant 6)

Another female reflected on her thoughts that people’s **compliance** with the strict measures would help her feel better:

*Maybe if all people would isolate themselves in their home, this would be helpful for me, because there are people who don’t abide by the measures and go out and unfortunately that’s how the virus is being transmitted* (Participant 8)

Notably, the same participant reported that there is **nothing** that could help her:

*I think there is nothing that could help me think even slightly differently about the coronavirus* (Participant 8)

Her view chimes well with that of another participant:

*No, I don’t have anything …* (Participant 4)

Two more adolescents could not think of any helpful practices at first; yet shortly after they were soon able to pinpoint specific ways through which others could help them feel better.

## Discussion

The aim of our research was to qualitatively investigate, through in-depth interviews, the perceptions, emotional reactions and needs of adolescent psychiatric inpatients amidst the COVID-19 pandemic. Preliminary knowledge about the complexity of subjective experience of this vulnerable category of adolescents, especially in the face of an unprecedented health crisis, will potentially enhance researchers’, clinicians’ and policy makers’ understanding of the patients’ perspective so that the former may develop tailored preventive and therapeutic interventions.

Almost all adolescents focused on information about the nature of coronavirus and on existing crisis management practices. Only one participant mentioned the probable psychosocial impact of the pandemic, and this may reflect a general trend to underestimate the ramifications of a mass disaster on mental health. Moreover, nearly all adolescent inpatients mostly identified negative changes due to the quarantine state. Considering that the subjects of the present study were adolescents (thus, individuals in a critical developmental phase during which a sense of autonomy must be safeguarded by all accounts [26]), the deprivation and restriction of freedom must not be overlooked. It should be noted here that the present research project offered insights by means of observing adolescents in two different types of treatment (inpatient and homebound). Most adolescents that remained hospitalized during the pandemic focused on their decreased contact with loved ones and the restriction of family visits, while some inpatients reported no significant changes. On the contrary, the group of adolescents who received homebound treatment reported further opportunities to spend more time with their family and to interact with others via technology. Indeed, such a practice was proved considerably useful for adolescents that received distance treatment. However, some homebound patients were ambivalent about this restriction. This finding may suggest that lockdowns and social distancing measures may confer an increased risk for adolescents’ mental health [4]. Thus, relevant issues, such as domestic violence and child abuse, may require a thorough evaluation in the event of any modification of the therapeutic settings [27].

In line with previous literature, adolescents in our research reported a wide array of pandemic-related negative thoughts (e.g., sadness, grief, anxiety and feelings of entrapment) [21]. Such adolescents’ thoughts and feelings may be triggered by the threat of harm to life and health, changes to daily life and human relationships or even by the reactions of family and social environment. Furthermore, these negative thoughts and emotions and pre-existing mental health problems may reciprocally influence each other [18]. That said, researchers and clinicians ought to focus on better understanding triggering and maintenance factors of pandemic-specific thoughts and feelings and shed light on the role of pre-existing mental health disorders (for example depression, anxiety and neurodevelopmental disorders [28].Our findings indicate that avoiding rumination about the coronavirus and its consequences, focusing on the positive, and looking towards the future were helpful strategies for coping with challenging feelings. Additionally, a sense of belongingness emerges as a key coping strategy. Trust in the work of authorities and the community was another particularly interesting point. Additionally, a balanced inflow and management of information, alongside the engagement in creative leisure activities and reaching out to others, were reported as beneficial. Moreover, our study indicates adolescents benefit not only from receiving balanced health messages, but also from a sensible, balanced mindset within their social and family environment. Accordingly, emotional regulation of the environment was deemed by adolescents as a highly helpful practice. A positive emotional atmosphere within the familial setting was the most reported helpful factor for many adolescents, and the same applied for shared leisure time activity as well as the joint planning of future activities whilst applauding the individuals’ adherence to the strict measures. What is more, it is proposed that parents become actively involved in the entirety of the therapeutic design, not least because the mental health state of parents quite often deteriorates alongside the worsening of their children’s behavioral health; in addition, there are cases where parents can address states of uncertainty and isolation while simultaneously helping their offspring cope with the new state of affairs due to COVID-19 [15, 16, 29, 30]. However, some adolescents did not acknowledge any helpful external intervention, thus reflecting traces of a feeling of helplessness.

According to our findings and previous recommendations, some suitable practices for both families and clinicians that will enable adolescents cope while in isolation may include: (a) establishing a regular routine and schedule at home, (b) helping adolescents keep in touch with friends and family members via technology, (c) keeping them informed about the current situation with honesty, using wording and concepts that are clearly intelligible, (d) helping adolescents find accurate and up-to-date information without overwhelming them, (e) developing a “shared understanding” within the family about coronavirus, and (g) making future plans [11]. Furthermore, adolescents mentioned various ways that help them cope with stress-ridden experiences amid the pandemic. Lending an ear to the patients’ voices and taking into consideration their experience will enlighten the scientific community about possible strategies to enhance patient resilience and prevent the aggravation of mental disorders whilst therapeutically addressing the patient needs during the pandemic.

Given all the aforementioned points, it would be feasible to argue in favor of the designing of personalized preventive and treatment interventions. Therapeutic targets should be based in the experiences of this extremely vulnerable population and facilitate sophisticated treatment planning decisions, such as staying hospitalized or sheltering at home. Given the fact that the role of the family and social environment in triggering, maintaining, and managing difficulties during this crisis proved instrumental in the present sample, their inclusion in these interventions cannot be overlooked.

Given the well-documented tendency to underestimate the impact of a mass disaster on mental health, it is essential for researchers to enhance the understanding of the pathways which may lead to adolescents’ stress-related disorders to design and evaluate personalized interventions [15, 31]. When it comes to hospitalized adolescents who are a priori considered as a population already burdened by severe psychopathology, the pandemic must be a serious wake-up call for researchers and policy makers to combat a possible mental health crisis in this vulnerable group [27, 32]. Finally, it is essential for clinicians who treat adolescents to engage in open discussions with both youths and their parents on the impact of the COVID-19 crisis on family life.

### Limitations

Τhe purpose of the present study was the collection of critical information on an unprecedented experience. It focused on a small sample of participants instead of seeking generalizable results. Consequently, the present findings do not aspire to reflect the whole range of perceptions and needs of the entire population. Moreover, future research should include in-depth interviews of parents and clinicians for the documentation of a variety of perspectives on youths’ experiences.

## Conclusion

Despite its limitations, the present study revealed important information about perceptions, feelings, and thoughts of adolescent psychiatric inpatients during the COVID-19 pandemic. To our knowledge, there is still no other qualitative research dealing with adolescent psychiatric inpatients. The latter constitute a particularly vulnerable population with greater mental health risks than outpatients or healthy adolescents. The information stemming from the present study may be proved useful for both researchers and decision-makers so that interventions that effectively address adolescents’ mental health problems within the context of hospitalization during the pandemic can be successfully promoted. Despite the extremely challenging circumstances, professionals should steadfastly carry on with providing both standard and emergency mental health care to mitigate any negative consequences for children and adolescents alike, adopting a more individualized orientation and making the most of the technological provisions in the digital era. Exhibiting flexibility and creativity in the clinical practice, including possible modifications of the therapeutic settings, may facilitate continuity of mental health care during all phases of the pandemic.

## Supplementary Information

**Additional file 1.** Open set of questions used in the study.

## Data Availability

The authors do not have the right to share any data information as per their institution policies.
