# Supplementary material for: Perceptions, emotional reactions and needs of adolescent psychiatric inpatients during the COVID-19 pandemic: a qualitative analysis of in-depth interviews
Source: BMC Psychiatry. 2021 Jul 28;21:379. doi: 10.1186/s12888-021-03378-w (PMC8316878; doi:10.1186/s12888-021-03378-w)
Supplement: Supplementary file 1 — Additional file 1. Open set of questions used in the study. [file 12888_2021_3378_MOESM1_ESM.docx]

**Open set of questions used in the study**

(a) What do you know about the new coronavirus?

(b) How has your daily life changed due to the coronavirus?

(c) How do you feel about what have been happening?

(d) Is there anything you can think or do to feel better?

(e) Is there anything other individuals can do to help you feel better?
